# Comparative Evaluation of Soda and Nitrate‐Alkaline Pulping of Hemp, Flax, and Sisal Post‐harvest Biomass

**DOI:** 10.1002/open.202500614

**Published:** 2026-03-20

**Authors:** Kateřina Hájková, Josef Bárta, Michaela Filipi, Adam Sikora

**Affiliations:** ^1^ Faculty of Forestry and Wood Sciences Czech University of Life Science Prague Prague Czech Republic; ^2^ Faculty of Chemical Technology University of Pardubice Pardubice Czech Republic

**Keywords:** nitrate‐alkaline pulping, nonwood pulp, rheosedimentation, soda pulping, tensile index

## Abstract

As wood is still scarce in some countries, it is necessary to replace wood in the paper industry with other lignocellulosic raw materials, particularly agricultural residues. This study evaluated hemp, flax, and sisal postharvest biomass as alternative nonwoody fibrous feedstocks for pulp and paper production. Pulping was performed using soda and nitrate‐alkaline methods under comparable degrees of delignification. Their suitability for papermaking was assessed in terms of mechanical properties, fiber sedimentation (rheosedimentation), and chemical parameters, including the degree of polymerization and the chemical composition of the raw material. The highest tensile strength was achieved for sisal, reaching 11.6 N m g^−1^ when produced by the soda method, which was higher than that of industrially produced flax or hemp soda pulps. Regarding pulp sedimentation behavior, hemp pulp showed significantly higher sedimentation rates, while comparable values were observed for recovered paper.

## Graphical abstract

The study compares hemp, flax, and sisal as nonwood raw materials for pulp production using soda and nitrate‐alkaline cooking. It evaluates chemical composition, cellulose crystallinity, yield, delignification, and paper strength. Sisal showed the highest tensile performance, while the choice of cooking method significantly influenced the quality and usability of the resulting pulps.

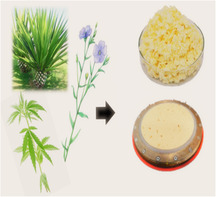

## 1 Introduction

Expanding the use of nonwoody fibrous raw materials as pulp for paper production could be crucial for today's paper industry, as there is a growing need to reduce wood consumption for pulp and paper production. Strategic efforts are therefore focused on identifying alternative lignocellulosic feedstocks for this large industrial field. Since the beginning of papermaking, nonwood materials have been used in the early stages of its development. Later, wood pulp became the dominant raw material; however, nonwood materials are again gaining attention, primarily due to limited wood availability in some regions.

In Europe, the utilization of nonwood materials for pulp and paper production is considered a way to substitute wood resources that can be allocated to higher value applications. An important advantage of nonwood materials is that they represent renewable feedstock available on an annual basis, depending on the plant species [1]. The rising interest in agricultural residues is global, as many studies have investigated their potential for pulp and paper production. In some countries, these feedstocks have already been implemented in industrial practice. For example, in India, agricultural residues contribute approximately 28% of the total feedstock used in the pulp and paper industry, mainly in the form of bagasse, wheat straw, rice straw, and cotton stalks [2].

From an environmental perspective, reusing postharvest residues in other products contributes to CO_2_ savings, as it avoids their disposal by open field burning or direct energy use, both of which may cause air pollution and related health issues. Therefore, research on postharvest residue utilization supports ecologically sustainable agriculture in Europe and may also be economically beneficial for local farmers [2]. In addition, consumers increasingly perceive packaging materials produced from agricultural residues as more environmental friendly [3].

According to the summary by Laberle and Westenbroek [4], the main arguments supporting the use of nonwood pulp for paper production are closely linked to sustainability, particularly in terms of regional circularity and the enhancement of local raw material supply. In addition, companies perceive nonwood pulp as an opportunity to diversify raw material sources, thereby increasing the resilience and flexibility of pulp and paper production systems.

Nonwoody fibrous raw materials can be theoretically divided into two groups according to Fišerová et al. [5]. The first group comprises low‐cost agricultural residues, such as straw, whereas the second group consists of more valuable long‐fiber materials suitable for special paper products and often more expensive than conventional wood pulp.

Although hemp and flax are traditionally associated with high‐value textile applications, this study focuses on postharvest residues rather than primary long bast fibers. Specifically, hemp, flax, and sisal were used in the form of secondary fibrous fractions remaining after primary processing, which contain shorter fibers but may still be suitable for pulp and paper production, particularly for specialty applications.

Each nonwood raw material requires a specific technological approach for pulping and subsequent paper production. Numerous studies have investigated various lignocellulosic materials, including postharvest residues, as alternative feedstocks for pulp production [5, 6, 7, 8, 9, 10, 11]. Previous research has confirmed that sisal (*Agave sisalana*) represents a suitable raw material for pulp production and subsequent paper products [8]. Hemp (*Cannabis sativa*) has also been extensively studied as a feedstock for pulp and paper production [12, 13, 14, 15]. For example, Gaynor et al. [16] used genetically improved hemp with fiber lengths of 1.0–1.2 mm and demonstrated that such material can substitute wood pulp in papermaking, producing fibers longer than those of conventional hemp and even birch pulp.

The final material investigated in this study, flax (*Linum usitatissimum*) postharvest residue, has likewise been evaluated in several studies [17, 18, 19, 20, 21]. Ramirez‐Cando et al. [20] demonstrated the suitability of flax as a feedstock for the pulp and paper industry using life cycle assessment, reporting a CO_2_ emission reduction of 450–1400 kg t^−1^ compared with conventional wood pulping.

The main advantage of nonwood pulp utilization lies in the diversification of raw materials available for pulp production. In Europe and other countries, where wood is increasingly used for higher value applications, such as construction and furniture manufacturing, agricultural residues represent a viable alternative feedstock for pulp and paper production. Such substitution could contribute to a reduction in overall wood consumption.

Moreover, studies focused on alternative pulp feedstocks are particularly relevant to developing countries that lack domestic pulping industries and therefore depend on imported pulp, despite having extensive agricultural land with crops that could serve as nonwood raw material. According to Haile et al. [22], nonwood pulp derived from Ethiopia's agricultural resources can partially replace wood‐based pulp. From a European perspective, however, the utilization of nonwood materials has shown a declining trend in recent years, and the full potential of nonwood feedstocks for pulp and paper production remains underexploited [23].

In this context, the present study aims to evaluate hemp, flax, and sisal postharvest residues as alternative nonwood raw materials for pulp and paper production, with particular emphasis on comparing soda and nitrate‐alkaline pulping methods under comparable degrees of delignification.

However, the technological application of individual nonwood raw materials can be challenging due to differences in their chemical composition and fiber morphology. In addition, logistical constraints may arise from the seasonal availability of postharvest residues, which can complicate their regular supply and transportation. Nevertheless, when appropriate pulping and processing strategies are applied, there is considerable potential to improve the utilization of nonwood materials in pulp and paper production. This study therefore seeks to expand current knowledge on the pulping potential of agricultural postharvest residues by systematically evaluating hemp, flax, and sisal using two different chemical pulping approaches.

## 2 Materials and Methods

### 2.1 Materials

This study used flax (*Linum usitatissimum*), hemp (*Cannabis sativa*), and sisal (*Agave sisalana*) as primary raw materials. Flax and hemp postharvest residues were obtained from local agricultural producers in the Czech Republic after fiber processing and consisted mainly of secondary fibrous fractions (shives). Sisal fibers were supplied by a commercial supplier from South Africa and were used as received.

In addition to the primary raw materials, industrially produced soda pulps from flax (Kappa number 19.86) and hemp (Kappa number 19.53) were supplied by the Delfort Group and used as reference materials for comparison with laboratory‐prepared pulps.

### 2.2 Chemical Analysis

Primary raw materials were first examined for their chemical composition, including macromolecular and low‐molecular‐weight components. Low‐molecular‐weight substances were determined as inorganic compounds in the form of ash according to TAPPI T 211 om‐02 [24], while organic extractives were determined as substances soluble in an ethanol–toluene mixture with a volume ratio of 7:3 according to TAPPI 280 pm‐99 [24]. Among macromolecular components, Seifert's cellulose [25] and Klason's lignin were determined according to TAPPI T 222 om‐02 [24]. All chemical analyses were performed in triplicate, and average values with standard deviations are reported.

In addition to determining the chemical components, the crystalline structure of cellulose was evaluated using Fourier transform infrared spectroscopy (FTIR). Prior to analysis, pellets with a diameter of 1.2 cm were prepared from the isolated cellulose using a Tempos TIRATEST 2580 press (TIRA GmbH, Schalkau, Germany) under pressure to a thickness of 1 mm.

The cellulose pellets were analyzed by FTIR with an accumulation of 64 interferograms at a resolution of 4 cm^−1^ in absorption mode over a wavelength range of 400–4000 cm^−1^, using a Nicolet iS20 FTIR spectrophotometer (Thermo Fisher Scientific Inc., Waltham, Massachusetts, USA). After baseline correction, several spectral parameters were calculated following deconvolution, including the total crystallinity index (TCI; absorbance ratio 1368/2895) and the amorphous region of cellulose (ARC; absorbance ratios 2895/1102 and 2895/3332). These indices were selected as commonly used semiquantitative indicators for evaluating changes in cellulose crystallinity and amorphous content in lignocellulosic materials.

### 2.3 Production of Pulp

#### 2.3.1 Soda Pulp

Sodium hydroxide was used as the cooking chemical at a concentration of 25% (w/w). Water was used as the cooking liquor with a liquor‐to‐solid ratio (hydromodule) of 5:1, and the active alkali charge was 19% based on oven‐dry raw material.

Cooking was performed in a laboratory batch digester and consisted of four successive stages. The process started with cooking to 105°C, followed by impregnation. Subsequently, the temperature was increased to 160°C, and cooking was completed to a total H‐factor of 1050 h. After cooking, the pulp was subjected to a four‐stage washing procedure involving dilution and subsequent thickening in order to remove residual cooking chemicals.

#### 2.3.2 Nitrate‐Alkaline Pulp

In the nitrate‐alkaline pulping method, nitric acid was used as the primary delignification agent at a concentration of 6% (w/w, based on oven‐dry material). During this stage, lignin was nitrated and partially oxidized to nitrolignin. Cooking was carried out at 95°C with a liquor‐to‐solid ratio of 10:1.

In the second stage, the nitrated lignin was removed by alkaline extraction using 5% (w/w) sodium hydroxide, followed by neutralization with 1% acetic acid and thorough washing with water. The combined duration of the acidic and alkaline stages was 45 min.

The nitrate‐alkaline method was selected as an alternative pulping approach due to its short processing time and relatively mild conditions, which may be advantageous for nonwood raw materials with lower lignin content.

### 2.4 Analysis of Pulp

The general properties of the pulps, namely, total yield and rejects content, were determined for soda and nitrate‐alkaline pulps produced from flax, hemp, and sisal. Rejects were separated using a laboratory flat screen equipped with a slotted sieve with a slot width of 0.20 mm. In addition, the kappa number according to ČSN ISO 302 [26], degree of polymerization, and sedimentation velocity were determined for these pulps as well as for the industrially produced soda pulps from flax and hemp used for comparison.

The degree of polymerization was determined using sodium tartrate with iron complexes and measured with an Ubbelohde capillary viscometer, according to SCAN‐CM 15:88 [27] and ISO 5351 [28]. The sedimentation velocity was determined using rheosedimentation, which is based on monitoring the kinetics of the spontaneous movement of the formed fiber network during sedimentation, following the method described by Milichovský [29].

### 2.5 Manufacturing of Laboratory Sheets

Laboratory handsheets were produced using a RAPID‐KÖTHEN RK‐2A laboratory sheet former (Birkenau, Germany) with a target basis weight of 80 g m^−2^. For each pulp sample, at least five handsheets were prepared to ensure reproducibility of mechanical testing.

The handsheets were subsequently conditioned at 23 ± 1°C and 50 ± 2% relative humidity in accordance with ISO 5269‐2 [30] prior to testing of mechanical and physical properties.

### 2.6 Mechanical Properties

Mechanical properties were evaluated by measuring the tensile properties according to ČSN EN ISO 1924‐2 [31] using a FRANK PTI tensile testing device (Birkenau, Germany). From the measured data, the tensile index was calculated.

In addition, the burst strength was measured and expressed as burst index using FRANK PTI laboratory equipment (Birkenau, Germany) in accordance with ISO 2758 [32].

## 3 Results

### 3.1 Chemical Analysis

**FIGURE 1:**
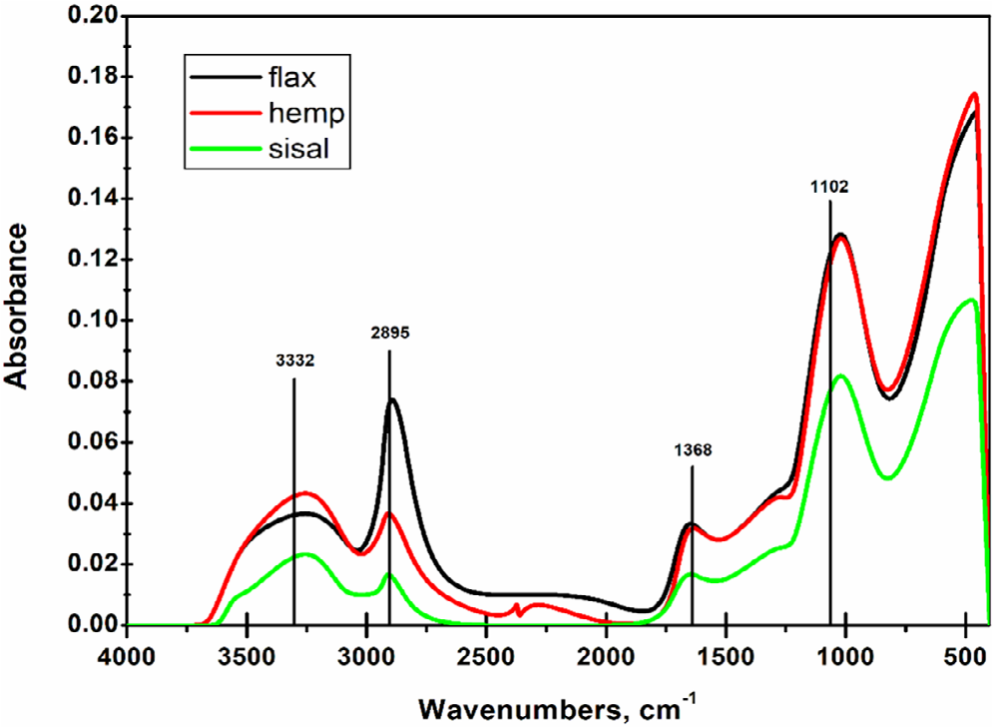
Representative FTIR spectrum of cellulose with marked absorbance bands used for calculating the total crystallinity index (TCI) and the amorphous region of cellulose (ARC).

Table 1 summarizes the average chemical composition of the investigated raw materials (flax, hemp, and sisal), while Figure 1 and Table 2 present the results of the FTIR analysis, including the crystalline and amorphous structure of cellulose.

**TABLE 1:** Chemical composition of flax, hemp, and sisal raw materials.

Raw/chemical components	Ash, %	Extractives, %	Cellulose, %	Lignin, %
Flax	2.94 (0.02)	1.52 (0.07)	39.17 (1.02)	25.08 (0.81)
Hemp	3.60 (0.03)	1.26 (0.04)	37.66 (1.63)	24.81 (1.39)
Sisal	1.38 (0.01)	0.19 (0.05)	43.17 (0.14)	19.65 (0.99)

*Note*: Values represent mean ± standard deviation (*n* = 3).

**TABLE 2:** Crystalline and amorphous cellulose indices determined by FTIR analysis.

Raw/FTIR components	TCI 1368/2895	ARC_1_ 2895/1102	ARC_2_ 2895/3332
Flax	2.0227	0.1350	0.5147
Hemp	1.4278	0.2993	0.5810
Sisal	1.0359	0.4543	0.9474

Abbreviations: TCI = total crystallinity index, ARC = amorphous region of cellulose, FTIR = Fourier transform infrared spectroscopy.

### 3.2 Analysis of Pulp

Table 3 summarizes the general properties of the cooked pulp, including rejects content, total yield, and Kappa number for soda and nitrate‐alkaline pulps produced from flax, hemp, and sisal. Table 4 presents the degree of polymerization for all pulp samples and reference cellulose, together with the corresponding pulp sedimentation velocities.

**TABLE 3:** General properties of soda and nitrate‐alkaline pulps produced from flax, hemp, and sisal.

General properties	Soda pulp			Nitrate‐alkaline pulp		
	Flax	Hemp	Sisal	Flax	Hemp	Sisal
Rejects content, %	1.04 (0.01)	0.60 (0.04)	0.71 (0.03)	0.91 (0.03)	0.74 (0.10)	1.14 (0.04)
Total yield, %	33.83 (0.47)	40.18 (1.07)	48.77 (1.19)	37.02 (0.10)	38.83 (0.55)	47.54 (0.44)
Kappa number	36.80 (0.15)	33.04 (0.73)	24.74 (1.21)	26.61 (1.07)	27.78 (0.86)	29.77 (1.13)

*Note:* Values represent mean ± standard deviation (*n* = 3).

**TABLE 4:** Degree of polymerization and standard sedimentation velocity of soda, nitrate‐alkaline, and industrial pulp.

Materials		Degree of polymerization	Standard sedimentation velocity, mm·s^−1^
Soda pulp	Flax	306	–
	Hemp	810	11.072
	Sisal	304	2.269
Nitrate‐alkaline pulp	Flax	226	4.292
	Hemp	238	–
	Sisal	211	4.531
Industrial soda pulp	Flax	533	4.473
	Hemp	560	5.232
Sisal	Flax	130	–
	Hemp	278	–
	Sisal	167	–

*Note:* The dash (–) indicates that sedimentation velocity could not be determined due to insufficient fiber network formation.

### 3.3 Mechanical Properties

**FIGURE 2:**
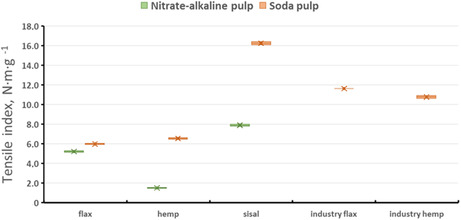
Tensile index of laboratory handsheets prepared from soda and nitrate‐alkaline pulps derived from flax, hemp, and sisal.

**FIGURE 3:**
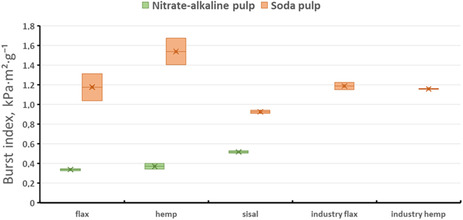
Burst index of laboratory handsheets prepared from soda and nitrate‐alkaline pulps derived from flax, hemp, and sisal.

The mechanical properties of the produced pulps, namely, tensile index and burst index, are presented in Figures 2 and 3, respectively. All laboratory handsheets were tested under standard conditioning conditions as described in Section 2.5.

## 4 Discussion

The chemical composition of the investigated raw materials is summarized in Table 1. Variations in chemical composition among nonwood raw materials have been reported previously and may result from differences in plant genotype, growing conditions, and climatic factors, as demonstrated by Fagbemigun et al. [31] for corn.

Among the investigated materials, the highest ash content was observed for hemp (3.6%), followed by flax, while sisal exhibited a considerably lower ash content (1.4%). These values are substantially lower than those reported for sunflower (8.2%) and corn (7.5%) [33]. In general, annual plants tend to exhibit higher ash than woody species due to increased mineral uptake and accumulation in nonwoody tissues. In comparison, significantly lower ash contents have been reported for woody plants, with approximately 0.5% for birch and 0.2% for pine [34].

The content of ethanol–toluene extractives determined for the investigated raw materials was lower than values reported for corn when using an ethanol–benzene system [35]. Furthermore, the extractive contents were also lower than those reported for beech (2.6%) and spruce (2.7%) [36]. This indicates that the studied raw materials contain a relatively low proportion of resinous and hydrophobic substances, which is advantageous for chemical pulping and subsequent papermaking processes.

The Seifert cellulose content of the analyzed raw materials was comparable to values reported in the literature for various agricultural residues. Similar cellulose contents have been reported by Housseinpour et al. [33] for corn (33.6%) and for wheat (38.2% for wheat), as well as for sugarcane trash (40.4%) [37]. Higher cellulose contents have been observed for rapeseed straw (49.9%) [38]. The values obtained in the present study were also comparable to those reported for wood species, namely, 41.0% for birch and 47.0% for pine, as reported by Barbash et al. [34].

Regarding lignin content, sisal exhibited the lowest lignin proportion (19.6%) among the investigated materials. Comparable lignin contents have been reported for other annual crops, such as wheat (15.3%), rapeseed (20.0%), corn (17.4%), sunflower (18.2%) [39], and sugarcane trash (17.4%) [37]. In comparison with woody plants, the lignin content of sisal was lower than that reported for deciduous species, such as oak (21.4%) and beech (24.5%) [36], while values for flax and hemp were closer to those of hardwoods. All investigated nonwood raw materials exhibited substantially lower lignin content than coniferous species, where lignin contents of 29.5% for pine and 30.4% for spruce have been reported [36].

The TCI and ARC were evaluated from absorbance peak height ratios using the methods described by Hrčka et al. [40]. Among the investigated raw materials, sisal exhibited the highest proportion of crystalline cellulose, with TCI values almost twice as high as those of flax and approximately 41.7% higher than those of hemp. However, these values are lower than those typically reported for woody species, such as spruce (TCI = 2.38) [41] and teak (TCI = 2.78) [42].

Chemical pulping is known to alter cellulose crystallinity, as the cooking process preferentially degrades hemicelluloses and less ordered cellulose regions [43]. The amorphous region of cellulose was evaluated based on the vibrational bands of secondary alcohol groups at 2895 and 1102 cm^−1^ and cellulose hydroxyl groups at 2895 and 3332 cm^−1^. As expected, flax exhibited the highest proportion of amorphous cellulose, while the ARC values were 45.7% and 38.7% lower for sisal and hemp, respectively. For comparison, ARC values of approximately 1.98 have been reported for teak wood [42].

During pulping, a further reduction in the amorphous cellulose structure can be expected, as penetration of the cooking liquor into the fibers leads to the exposure of the microfibril bundles and preferential removal of amorphous regions located on the surface of the macrofibrils. This structural change facilitates delignification and contributes to improved accessibility of lignin during chemical pulping [44].

Pulp properties were evaluated for both soda and nitrate‐alkaline pulps, with particular focus on rejects content, total yield, degree of polymerization, and sedimentation behavior. For soda pulping, the lowest reject content was observed for hemp pulp (0.60%), while the highest reject content was recorded for flax pulp (1.04%). In the case of nitrate‐alkaline pulping, hemp again exhibited the lowest rejects content (0.74%), whereas sisal showed the highest value (1.14%).

Regarding total pulp yield, the highest yield was obtained for sisal soda pulp (48.8%), while the lowest yield was observed for flax soda pulp (33.8%). For comparison, lower yields have been reported for rice straw pulped by the soda method (39.0%), whereas yields similar to those obtained in the present study have been achieved for wheat straw and for sisal (46.7%) [45]. In contrast, substantially higher yields have been reported for chemo‐thermomechanical pulping of rapeseed (90.3%) and the thermomechanical pulping of pine (94.0%) [38]. These higher yields result from incomplete delignification and limited chemical removal of noncellulosic components, leading to higher pulp mass but lower fiber purity.

The degree of polymerization (DP) was evaluated in comparison with industrial soda pulps and reference cellulose. The highest average DP was obtained for hemp soda pulp (810), whereas the lowest DP was observed for flax cellulose (130). Lower DP values observed for nitrate‐alkaline pulps indicate partial cellulose degradation during the rapid acidic–alkaline treatment, which may adversely affect fiber strength.

The sedimentation velocity could not be determined for all samples, as in some cases the fiber network formation was insufficient, or the sedimentation rate was too high for accurate measurement. The highest measured sedimentation velocity was observed for hemp soda pulp (11.07 mm s^−1^), while the lowest value was recorded for flax nitrate‐alkaline pulp (4.29 mm s^−1^). The sedimentation behavior of the investigated pulps was superior to that reported for A5 (2.35 mm s^−1^) and A6 (2.40 mm s^−1^) grade recovered paper but did not reach the sedimentation velocity of sulfite pulp (7.33 mm s^−1^) [46].

The primary objective of this study was to evaluate the mechanical properties of paper produced from flax, hemp, and sisal postharvest residues using two chemical pulping methods. As expected, the highest mechanical performance was achieved for industrially produced soda pulps, which represent optimized reference materials. Nevertheless, laboratory‐scale pulping yielded promising results, particularly for sisal soda pulp and flax pulp produced by the nitrate‐alkaline method.

The tensile index values obtained in this study cannot be directly compared with those of kraft pulps from coniferous wood, where tensile indices as high as 78.40 N m g^−1^ have been reported [47]. Such high values are typically achieved through optimized industrial processing and fiber refining and therefore exceed the scope of the present work. Accordingly, the mechanical properties were compared primarily with pulps derived from annual plants and agricultural residues.

Amode and Jeetah [13], who investigated Mauritian hemp pulped by the soda method, reported a tensile index of 10.97 N m g^−1^, which is comparable to the values obtained in the present study. Bosco et al. [8] reported a tensile index of 9.90 N m g^−1^ for sisal pulp, which is lower than the tensile index achieved for sisal soda pulp in this work. Fišerová et al. [5] reported tensile indices of 26.10 N m g^−1^ for soda pulp from rice straw and 3.20 N m g^−1^ for corn pulp, highlighting the strong influence of raw material and processing conditions.

Higher tensile indices have been reported for rapeseed pulp produced by chemo‐thermomechanical pulping (CTMP), reaching 83.2 N m g^−1^ [38]. However, such values result from fundamentally different pulping mechanisms, limited lignin removal, and intensive mechanical treatment and therefore cannot be directly compared with the chemically pulped, unbeaten laboratory pulps investigated in this study. All handsheets in the present work were prepared from unbeaten pulps to evaluate the intrinsic fiber properties and the influence of pulping chemistry rather than process optimization.

Burst index is strongly affected by fiber bonding and refining intensity, and therefore low values are typically reported for unbeaten pulps from agricultural residues. For example, unrefined wheat straw soda pulps commonly exhibit burst index values around 1–2 kPa m^2^ g^−1^, whereas beating or optimized pulping can increase these values to approximately 4–6 kPa m^2^ g^−1^, as reported by Ahmed et al. [12] and Deniz et al. [48] Similar trends have been observed for rapeseed straw pulps, where soda and soda–AQ pulps show low burst indices prior to refining, followed by significant improvement after fiber treatment [49, 50]. The burst index values obtained in the present study therefore represent baseline pulp performance. Lower burst indices observed for nitrate‐alkaline pulps are consistent with their lower degree of polymerization, which limits inter‐fiber bonding.

## 5 Conclusion

This study demonstrated that the pulping method significantly influences the mechanical properties of pulps produced from postharvest residues of annual plants. Differences between soda and nitrate‐alkaline cooking were observed across all investigated raw materials, confirming the importance of selecting an appropriate delignification strategy for nonwood fibers.

The lowest tensile index values were obtained for nitrate‐alkaline hemp pulp, while flax and hemp pulps produced by both cooking methods exhibited generally moderate tensile properties. In contrast, sisal soda pulp achieved the highest tensile index among the laboratory‐prepared pulps investigated, whereas nitrate‐alkaline sisal pulp exhibited distinctly lower tensile performance. Compared with industrially produced flax or hemp pulps, sisal demonstrated considerable potential as an alternative nonwood fiber source for pulp and paper production.

Future research should focus on optimizing cooking conditions, including variation of cooking time, degree of delignification, and posttreatment strategies. In particular, the incorporation of oxidative agents such as peroxyacetic acid may improve optical properties, including pulp brightness, and further enhance the applicability of nonwood pulps for paper produced.

## Author Contributions

**Kateřina Hájková:** conceptualization (lead), formal analysis (lead), investigation (lead), methodology (lead), project administration (lead), supervision (lead), validation (lead), visualization (lead), writing – original draft (lead), writing – review & editing (lead). **Josef Bárta:** formal analysis (equal), investigation (equal), methodology (supporting), validation (supporting), writing – original draft (supporting), writing – review and editing (supporting). **Michaela Filipi:** formal analysis (supporting), validation (supporting). **Adam Sikora:** methodology (supporting), visualization (equal), writing – original draft (supporting).

## Funding

The authors have nothing to report.

## Conflicts of Interest

The authors declare no conflicts of interest.

## Data Availability

Dataset available on request from the authors: The raw data supporting the conclusions of this article will be made available by the authors on request.
